# Best practices for first psychedelic experiences: harm reduction advice from the psychedelic community

**DOI:** 10.1186/s12954-025-01337-2

**Published:** 2025-11-25

**Authors:** Daniel J. Kruger, Gina Mersereau, Ashley Sullivan, Julie Barron, Moss Herberholz, Niloufar Pouyan, Jacob S. Aday, Kevin F. Boehnke

**Affiliations:** 1 Department of Community Health and Health Behavior, School of Public Health and Health Professions, University at Buffalo, 3435 Main Street, Buffalo, NY 14214 USA; 2 Michigan Psychedelic Center, University of Michigan Medical School, 1301 Catherine Street, Ann Arbor, MI 48109 USA; 3 Jacobs School of Medicine and Biomedical Sciences, University at Buffalo, 955 Main Street, Buffalo, NY 14203 USA; 4 Warner School of Education and Human Development, University of Rochester, 500 Wilson Boulevard, Rochester, NY 14627 USA; 5 Blue Sage Health Consulting, 1829 W Stadium Blvd # 700, Ann Arbor, MI 48103 USA; 6 Michigan Psychedelic Society, 1829 West Stadium Blvd, Suite 700, Ann Arbor, MI 48103 USA; 7 The Radical Well-Being Center, 20411 W 12 Mile Rd Suite 101, Southfield, MI 48076 USA; 8 Anesthesiology Department, University of Michigan Medical School, 24 Frank Lloyd Wright Drive, Lobby M, Ann Arbor, MI 48105 USA; 9 Neuroscience Graduate Program, University of Michigan Medical School, 204 Washtenaw Ave., Ann Arbor, MI 48109 USA

**Keywords:** Psychedelics, Adverse experiences, Harm reduction

## Abstract

**Background:**

The use of psychedelics is currently increasing in the United States. Awareness of clinical trials investigating the therapeutic applications of psychedelics may result in a record number of people who use psychedelics for the first-time. This study aimed to develop a harm-reduction resource to facilitate safe and successful psychedelic experiences outside of regulated clinical and research settings. We employed a community-based approach to crowdsource practical recommendations for first-time psychedelic experiences from the psychedelic community.

**Methods:**

We conducted an online survey with 581 individuals who reported psychedelic use (*N* = 581) on recommendations for people using psychedelics for the first-time, following the principles of community-based collaborative research. The survey assessed recommendations for and against specific psychedelics for first-time experiences, recommendations for and against combinations of psychedelics, and other advice for first-time experiences. Open-ended follow-up questions were included to understand participants' reasons for their recommendations. An experienced qualitative researcher and two qualitative coders analyzed responses to open-ended items.

**Results:**

Most participants recommended psilocybin for first-time psychedelic experiences, approximately half recommended cannabis, and a third recommended MDMA/MDA (3,4-methylenedioxymethamphetamine/3,4-methylenedioxyamphetamine, ecstasy, molly). These substances were favored for their moderate intensity, dose-dependent effects, precise dosing, and relatively short duration of effects. Conversely, substances such as ayahuasca, DMT (*N*,*N*-dimethyltryptamine), 5-MeO-DMT (5-methoxy-*N*,*N*-dimethyltryptamine), and *Salvia divinorum* or salvinorin A were not recommended due to their intensity, mental and physical health risks, and safety concerns. Participants advised against mixing psychedelics with alcohol, stimulants, antidepressants, and narcotics/opiates. Additional recommendations included embracing the experience, learning about the substance and its effects, and setting intentions for the experience.

**Conclusions:**

Given the growing interest in psychedelics despite limited legal access and systematic education available, it is crucial to inform the public about practices that minimize risks. This project compiled recommendations from individuals who self-identified being experienced with psychedelics. The active involvement of the psychedelic community may enhance research quality and public trust in the findings.

**Supplementary Information:**

The online version contains supplementary material available at 10.1186/s12954-025-01337-2.

## Background

Psychedelic substances are serotonergic hallucinogens, some of which have been used for millennia in various cultural and religious contexts worldwide [1]. During the mid-twentieth century, psychedelic research flourished, demonstrating substantial potential for treating conditions such as depression and substance addiction [2]. However, promising research programs were discontinued during the culture wars of the late 1960s when psychedelics were criminalized with the initiation of the War on Drugs and the accompanying moral panic around these substances [3, 4]. Despite this, there continued to be considerable levels of illicit psychedelic use and underground research [2].

There has recently been a resurgence of interest in psychedelics, with both classic psychedelics (5-HT2A agonists, e.g., psilocybin, Lysergic acid diethylamide (LSD)) and non-classic psychedelics (psychoactive substances with other neuropharmacological mechanisms; e.g., MDMA, ketamine, ibogaine) being studied for potential mental health benefits, particularly when paired with psychotherapy [5, 6]. Pharmaceutical companies and venture capitalists are investing billions of dollars in clinical trials [7]. As of January 2025, there were at least 73 registered clinical trials studying the potential therapeutic efficacy of psilocybin alone [8]. Reports in respected news outlets and popular books like Michael Pollan’s *How to Change Your Mind* [9] have raised public awareness of these substances and their potential benefits. Legislative changes have liberalized psychedelic access for personal use [10] and institutional health care [11]. Not surprisingly, levels of psychedelic use are increasing in the general population and may be at all-time highs [12, 13].

Although psychedelic-assisted psychotherapy is becoming increasingly accessible, most classic psychedelic use continues to happen outside of a regulated context. Rather than waiting for legal medical access to become available, many individuals are choosing to use psychedelics by other means [14, 15]. Decriminalization (or de-prioritization by law enforcement) may increase accessibility but does not guarantee oversight. Two U.S. states have legalized psychedelic therapy, though individual sessions at licensed psilocybin centers in Oregon range from $1,500 to $3,200 [16], and a full course of therapy is estimated to cost between $7,638 and $9,532 [17]. There is no health insurance coverage for these services, which limits access to those with sufficient resources to pay out of pocket. Optimistic expectations were tempered by the Food and Drug Administration’s (FDA) recent decision to reject Lykos Therapeutics' New Drug Application for MDMA combined with psychotherapy to treat posttraumatic stress disorder. There will be a longer wait for broader legal access to psychedelics, which may happen eventually, given that Lykos Therapeutics is initiating another Phase 3 clinical trial to address FDA concerns and there are hundreds of other active clinical trials. There is considerable media attention to the therapeutic clinical trials intended to facilitate the approval for the prescription of new pharmaceutical products. Recreational psychedelic use has been associated with better mood, higher social connectivity, and transcendent experiences [18].

## Adverse experiences

Most classic psychedelics are generally considered to have fewer physiological adverse effects compared to legal recreational and pharmaceutical drugs [4, 19]. However, adverse experiences including suicidality, psychotic disorder, manic symptoms, cardiovascular events, and hallucinogen persisting perception disorder have been documented in clinical or research administrations of psychedelics [20], and some researchers have noted that risks are somewhat understudied in the face of increasing hype around psychedelic benefits [21]. These include psychological adverse events and serotonin syndrome when certain psychedelics are taken by those using specific antidepressant classes [22]. The enthusiasm for the potential therapeutic applications of psychedelics may have led both researchers and patients to downplay adverse effects [23], an important area which is currently understudied [18]. Concerns about inadequate assessment of adverse events were highlighted in the Institute for Clinical and Economic Review report regarding the Lykos application [23]. Recently, there has been increasing attention among psychedelic researchers to better characterize the risks and potential adverse effects of psychedelics [24]. In a large survey of people who have used ayahuasca, most reported adverse physical and mental health effects [25]. Vomiting was the most frequent adverse physical effect, with some respondents experiencing headaches, abdominal pain, and breathing difficulties; about 2% sought medical treatment after their experience [25]. Adverse mental health effects included feelings of loneliness, nightmares, anxiety, uncontrollable worrying, and derealization; around 12% sought professional support afterward [25]. Most individuals who have experienced a broad range of psychedelics report adverse experiences such as fear, sadness, body tremors, and loneliness [26]. About half reported experiencing panic, despair, and fearing that their current state would last forever [26]. Smaller but substantial proportions reported feeling as if they were dead or dying, fearing they would lose their mind, and paranoia [26]. Those who consume psychedelics at musical parties or festivals have experienced hallucinations (e.g., hearing voices), disorientation, and derealization [27]. Adverse experiences such as anxiety and fear, existential struggle, social disconnection, depersonalization, and derealization can persist after psychedelic experiences, sometimes for over a year [28]. Without the availability of a legal and regulated option for therapeutic use, risks are increased for those who seek to use psychedelics therapeutically but are unsure of how to do so safely [14].

## Psychedelics and harm reduction

The widespread interest in and use of psychedelics outside of regulated environments, combined with the potential for adverse experiences and events, necessitate a harm reduction approach. U.S. drug policy has historically focused on reducing drug use and making drugs less available, though in the 1990s public health researchers argued for a shift towards reducing the harms to society [29, 30]. The Mersey Harm Reduction Model, created in Liverpool, England in the mid-1980s in response to the emergence of HIV/AIDS [30], shifted the focus from exclusively reducing drug use to reducing the risk of harms from drug use [30], with goals of reducing overdose rates and limiting the spread of infectious diseases [31]. Harm reduction is now recognized as a critical evidence-based approach for engaging with those who use drugs by the Substance Abuse and Mental Health Services Administration [32] and is also a key aspect in the U.S. Department of Health and Human Services' Overdose Prevention Strategy [32].

Harm reduction is broadly relevant to all personal use of any substance, acknowledging personal autonomy and reducing risk and maximizing benefits from personal choices [14]. Harm reduction programs do not increase drug use [33]. Some argue that because the potential benefits of psychedelics compare favorably to the potential harms in comparison to other psychoactive substances, a harm reduction approach may not only focus on safety but also facilitate the potential benefits of psychedelics [14, 15]. Drug testing is one form of harm reduction, though these services are prohibited in some countries [34]. Guidelines and recommendations have been created for carefully controlled psychedelic research and clinical settings [14, 35, 36].

The use of psychedelic harm reduction practices is positively associated with emotional breakthroughs [15], a key mediator of longer-term positive psychological changes [37], and is negatively associated with challenging experiences [15]. Notably, individuals use fewer harm reduction practices in their first experiences, employing more and a wider range of harm reduction practices as they gain experience [15]. These practices include ensuring a comfortable setting, setting a purpose, obtaining the drug from a reputable source, measuring the dose, and arranging a time to take the drug [15].

Psychedelic use outside of carefully controlled environments with therapeutic support may increase the risk of difficult or adverse experiences [15], though even therapeutic environments can have adverse events such as inappropriate sexual contact [38]. Harm reduction advice for psychedelic use outside of regulated environments largely consists of catchphrases that may resonate with those who have experienced psychedelics but may perplex the uninitiated. “Turn off your mind, relax, and float downstream…surrender to the void,” sang the Beatles [39], paraphrasing Leary, Metzner, and Alpert’s *The Psychedelic Experience*, [40] adapted from the *Tibetan Book of the Dead*, [41] a Western interpretation of *The Great Liberation upon Hearing in the Intermediate State*. The original Tibetan Buddhist texts were meant to be read aloud to the deceased to help guide their consciousness to a favorable next life [2]. Phrases such as “surrender to the void” [39] may be loaded with meaning for the psychedelic community, though even with elaboration people may not have a concrete idea of what exactly they should plan or do.

## Current study

Given the widespread interest in psychedelics, limited options for legal access, and lack of systematic education, it is critical to inform the public of practices which minimize the risk of harms of psychedelic use. We believe that minimizing the risk of harm from psychedelic use will also help to promote the benefits of psychedelic use, though the relationship between risk minimization and benefit maximization may be complex and is an important question for future research. The knowledge and experiences of those who already use psychedelics may be a valuable resource, though it is difficult to access this information without personal connections. There is a need to investigate harm reduction beliefs and practices in psychedelic using populations [34] and to communicate effective practices to those using psychedelics for the first time [15]. In the current project, our objective was to crowdsource practical recommendations for first psychedelic experiences from people currently using psychedelics, creating a practical and legitimate resource for first-time psychedelic experiences. We recruited individuals who have knowledge regarding harm reduction practices derived from personal experiences with psychedelics and recruited through professional networks to include individuals with both personal experience and professional roles related to psychedelics (e.g., therapist, sitter, researcher). We hypothesized that participants would emphasize preparing for the substance use, using substances that were considered less psychologically and physically dangerous, and including practical tips like substance testing and starting with low doses.

## Methods

We employed a community-based approach [42–45] to conduct a survey assessing various aspects of psychedelic use in a multi-component survey. Our team, comprising academic researchers, psychedelic therapists, and community leaders, collaboratively designed survey items to gather recommendations for first-time psychedelic experiences from the psychedelic community. We recognize that the “psychedelic community” is not a unified entity, though the aggregation of advice across many individuals and roles regarding psychedelics will reveal important patterns and trends. We conducted a large-scale survey following the principles of community-based collaborative research [42–45]. This approach included the involvement of members of the psychedelic advocacy community in every stage of the project and consensus decision making. A collaborative research environment where community members actively participate can produce academically rigorous work that is also socially responsive and beneficial to the communities involved. The active involvement of psychedelic community members may enhance the quality of research and increase public trust in the results.

Participants were recruited in person at Entheofest, a psychedelic advocacy event in Ann Arbor, MI, on September 17, 2023, and via psychedelic-related email listservs, newsletters, and social media platforms (e.g., Reddit, Facebook) from September 17 to October 31, 2023. The “Prevent Ballot Box Stuffing” setting in Qualtrics was used to prevent duplicate responses. Adults aged 18 years and older who had taken a psychedelic substance and had not previously completed the survey were eligible to participate.

### Survey design

The survey included seven questions regarding recommendations for people experiencing psychedelics for the first-time. Participants were first asked “Are there psychedelic(s) you would recommend for a first experience? (Select all that apply)” and were provided with a list of 20 substances or classes of substances, e.g., “Synthetic tryptamines (AL-LAD, ETH-LAD, 4-HO-MET, 5-MeO-MiPT, etc.)” Participants were also provided with a “No/None” option and a “Other:” option with an open-ended text box. We included “classic” psychedelics such as LSD and psilocybin, which are 5HT2A agonists, non-classic psychedelics such as MDMA and Ketamine, and substances which are often grouped with non-classic psychedelics such as cannabis (THC) [46]. Wittgenstein argued that a term's use *is* its meaning [47], and researchers have incorporated this approach into psychedelic realms, acknowledging that how people actually use these terms in naturalistic language may be more important than pharmacological definitions in some contexts [48]. We took a similar inclusive approach, as cannabis was considered to be a psychedelic by over a third of respondents in a recent international survey of over 8000 individuals experienced with psychedelics [49].

Those who selected any psychedelic substance or class of psychedelic substances were asked, “Why would you recommend this/these for a first experience?” Subsequently, participants were asked “Are there psychedelic(s) you would NOT recommend for a first experience? (Select all that apply)” with the same response options and a parallel follow-up question, “Why would you NOT recommend this/these for a first experience?” These items were followed by open-ended text items, “Are there psychedelics that you recommend taking together? (or other substances to take with psychedelics?)”; “Are there psychedelics that you DO NOT recommend taking together? (or other substances NOT to take with psychedelics?),” and “What other recommendations do you have for someone using psychedelics for the first time?”.

### Data analyses

Frequencies were computed for psychedelics recommended and not recommended for a first experience. A table of all possible binary combinations of psychedelics was used to document recommendations for and against psychedelic combinations, with a numeric code for each combination. Recommendations not to mix psychedelics were coded with a list of substances (e.g., alcohol).

An experienced qualitative researcher (DJK) and two qualitative coders (GM, AS) analyzed responses to open-ended items. The content was reviewed and discussed to generate themes and sub-themes for each item. Coders independently coded a subset of responses for each item, and the team collectively revised the coding scheme based on coder feedback. Coders independently coded responses to each item using the final coding scheme. Coders indicated all applicable codes for each response. Discrepancies were discussed and resolved, resulting in a consensus set of codes used for analyses. The overall coding discrepancy rate was 4.2% (825/19425); all discrepancies were successfully resolved.

Recommendations and other content that was outside of the scope of an item’s content (e.g., “be with people you love and trust” in response to “Why would you recommend this/these for a first experience?”) were combined with the responses to “What other recommendations do you have for someone using psychedelics for the first time?” for each participant. Thus, these recommendations were documented per participant, regardless of where in the survey the participant entered the advice. Similarly, those who explicitly advised against combining psychedelics in response to “Are there psychedelics that you recommend taking together?” were included in the proportion of participants computed for “Do not mix any psychedelics” in “Recommended to not combine.” Participants who responded “No,” “Not really,” were coded as “No recommendation.” Those who indicated a lack of knowledge or experience (e.g., “Not enough experience to comment.”) were coded as “Unsure, do not know.” Participants who did not provide a response were not included in these proportions. Some participants gave responses without specific content, e.g., “Have fun, man,” “You covered them well. Good prep for the journey,” these responses were not coded. There were no responses containing any suggestions deemed to be harmful advice.

Chi-squares tested whether those having experiences with specific substances were more likely to recommend them for a first psychedelic experience or recommend not to use them for a first psychedelic experience. These analyses were conducted for the top five psychedelics recommended and not recommended for a first experience. We calculated the total number of unique pieces of advice given by each participant. We did not include the number of psychedelics recommended or not recommended in these totals, though we did include the reasons given for making these recommendations. We did not include responses indicating that the participant was unsure or did not have enough knowledge or experience to provide advice. We conducted a stepwise regression analysis predicting the total number of unique pieces of advice given. Candidate predictors included age in years, education level in years, income in USD, White versus Non-White ethnicity, whether participants identified as a woman, whether participants identified as gender diverse (non-binary, transgender, etc.), frequency of psychedelic use in the past five years, number of different psychedelics used (lifetime), and whether participants had experienced full doses of psychedelics or only microdoses.

## Results

### Participant characteristics

Participants (*N* = 581) reported hearing about the survey via email (40.8%), social media (27.2%), the Entheofest event (12.6%), other sources (17.7%; newsletters, etc.), with 1.7% of participants not answering this item (See Table 1 for Sociodemographic characteristics).

**Table 1:** Participant descriptives (*N* = 581)

*Descriptive*	
Gender	
Woman	50.8%
Man	44.2%
Non-binary	2.9%
Trans-Woman	0.7%
Trans-Man	0.3%
Other identity	0.7%
(Missing)	0.3%
Age in years (M, SD, range)	44, 15, 18–85
Education in years (M, SD, range)	16, 2, 10–20
High school graduate, GED, or less	4.3%
Some college, technical, or associate’s degree	26.5%
Bachelor’s Degree	26.5%
Master’s, Doctorate or Professional Degree	42.2%
Races/ethnicities (inclusive)	
Caucasian/White	78.7%
Hispanic/Latino	4.6%
African American/Black	3.6%
Asian	3.3%
Aboriginal/First Nation/Native American	3.1%
Native Hawaiian/Pacific Islander	0.2%
Other	1.4%
Frequency of psychedelic use in the past five years	
Not in the past 5 years	4.5%
Once	9.1%
Once per year	11.2%
Once every 6 months	15.0%
Once every 2–5 months	27.9%
Once every month	15.8%
Once every week	5.5%
More than once per week	11.0%
Roles in relation to psychedelics (inclusive)	
User	57.0%
Researcher—Personal	43.0%
Psychonaut	39.4%
Guide/Sitter	33.2%
Activist (involved in specific organizations)	29.3%
Grower/cultivator	23.8%
Therapist	20.8%
Educator	20.0%
Healthcare provider	16.7%
Researcher—Academic	13.1%
Student (in training for psychedelic therapy)	12.7%
Shamanic practitioner	11.5%
Coach	11.0%
Entrepreneur	10.7%
Author	9.8%
Indigenous practitioner (participant)	8.3%
Chemist	7.1%
Indigenous practitioner (facilitator)	6.0%
Journalist	4.3%
Researcher—Industry	4.3%
Trainer	4.3%
Clergy	2.8%
Policymaker	2.8%

Participants most frequently reported using psilocybin mushrooms or truffles (91.9%), lysergic acid diethylamide (LSD, acid; 43.9%), MDMA/MDA (Ecstasy, Molly; 40.4%), and Ketamine (28.1%). Most (85.4%) had taken full doses of psychedelics (effects are clearly or strongly felt), whereas 14.6% had only taken microdoses of psychedelics (effects are sub-perceptual; these definitions were provided to participants). Most participants (80.8%) had experienced at least two different psychedelics. Additionally, 86.4% had taken psychedelics in the past year, 75.2% had taken psychedelics at least twice in the past year, and 4.5% had taken psychedelics but not in the past five years.

### Substances recommended for first-time psychedelic experiences

Approximately three-quarters of participants recommended psilocybin mushrooms or truffles for first psychedelic experiences (Table 2). No other substance was recommended by most participants. About half recommended cannabis, and about a third recommended MDMA/MDA for first psychedelic experiences. Participants recommended these substances primarily due to desirable characteristics, such as not being too intense, having dose-dependent effects and the ability to be dosed precisely, and having a short duration of effect (Table 3). The next most common theme was positive effects, such as cognitive enhancement and empathogenic or prosocial effects. Participants also cited the existing knowledge on the substance as a reason for recommendation, the third most common theme. About one in ten participants expressed that their recommendations depended on the set and setting or the person taking the substance. Those who had experience with the top five recommended substances were more likely to recommend them for a first psychedelic experience: Psilocybin mushrooms or truffles (77.7% vs. 27.7%, χ^2^_(1)_ = 55.79, *p* < 0.001), cannabis (60.2% vs. 17.8%, χ^2^_(1)_ = 86.51, *p* < 0.001), MDMA/MDA (58.3% vs. 11.6%, χ^2^_(1)_ = 144.31, *p* < 0.001), LSD (33.7% vs. 4.9%, χ^2^_(1)_ = 82.09, *p* < 0.001), and Ketamine (33.1% vs. 5.0%, χ^2^_(1)_ = 82.39, *p* < 0.001).

**Table 2:** Psychedelics recommended and not recommended for a first experience (*N* = 581)

Substance	% Recommending	% Not recommending
Psilocybin (mushrooms or truffles)	73.7	2.6
Cannabis	47.8	2.1
MDMA/MDA (Ecstasy, Molly)	30.5	6.0
Lysergic acid diethylamide (LSD, acid)	17.6	17.9
Ketamine	12.9	12.6
Mescaline (Peyote, San Pedro cacti)	6.2	11.7
Psilocybin (synthetic)	6.0	5.7
Nitrous oxide	5.7	7.9
DMT	3.3	28.2
Synthetic phenethylamines	2.8	15.5
Ayahuasca	2.6	29.3
Lysergic acid amide (LSA, Ergine)	1.2	9.3
*Amanita muscaria* (fly agaric)	1.0	15.5
5-MeO-DMT	1.0	19.8
Dextromethorphan (DXM)	0.9	11.2
Kambo	0.9	9.6
Synthetic tryptamines	0.9	14.5
*Salvia divinorum* or salvinorin A	0.7	18.8
Bufotenin	0.5	11.4
Iboga/Ibogaine	0.2	15.5

**Table 3:** Reasons for recommending specific psychedelics (*N* = 581)

Major themes	%
Desirable characteristics of substance	69.4
Positive effects	28.9
Existing knowledge on substance	16.7
Availability/Accessibility*	15.3
Therapeutic indications*	12.0
It depends	10.5
Desirable characteristics of substance	69.4
Not too intense	30.4
Experience is dose dependent and can be dosed precisely	25.4
Short duration	13.4
Safe	8.1
Natural/organic	7.4
Can be microdosed	5.6
Unlikely to have a bad experience	3.5
Lack of negative side effects	2.3
Reliable purity	2.1
Predictable trajectory/experience	1.9
Inability to overdose	1.7
Powerful	1.6
Ability to self-guide	1.4
Long duration	1.0
Not addictive	1.0
Fast onset	1.0
Favorable/predictable duration	0.8
Few GI issues	0.8
Lack of residual effects	0.6
Slow onset	0.6
Few contraindications	0.4
Ecological sustainability	0.2
Positive effects	28.9
Cognitive enhancement	8.5
Empathogenic/prosocial	7.4
Calming/peaceful	3.7
Euphoria	3.3
Spiritual	1.0
Beautiful	1.0
Ego death/dissolution	0.6
Clean/pure effect	0.4
Existing knowledge on substance	16.7
Personal experience	12.2
Science/research	2.1
History/cultural traditions/indigenous practices	1.7
It depends	10.5
Depends on set and setting	6.2
Need the right fit for person	3.5
All are OK in the right circumstances	2.3

*No sub-themes

### Substances not recommended for first-time psychedelic experiences

Participants most frequently advised against using ayahuasca (29.3%), DMT (28.2%), 5-MeO-DMT (19.8%), and *Salvia divinorum* or salvinorin A (18.8%, Table 2) for a first psychedelic experience. Only 2.6% of participants recommended not to use psilocybin mushrooms or truffles for first psychedelic experiences. Participants recommended not to use these substances for a first experience primarily due to undesirable characteristics (Table 4), such as high potency/being too intense and having a long duration of effect. Mental health risks and safety concerns were the next most common theme, followed by physical health risks and safety concerns. Finally, participants did not recommend substances due to limited knowledge and information, especially when participants had no personal experience with the substance. Those who had experience with the top five substances NOT recommended were more likely to NOT recommend them for a first psychedelic experience: Ayahuasca (39.7% vs. 27.9%, χ^2^_(1)_ = 4.06, *p* = 0.043), DMT (49.5% vs. 23.3%, χ^2^_(1)_ = 18.57, *p* < 0.001), 5-MeO-DMT (37.8% vs. 18.6%, χ^2^_(1)_ = 8.11, *p* = 0.004), *Salvia divinorum* or salvinorin A (51.1% vs. 16.0%, χ^2^_(1)_ = 33.49, *p* < 0.001), and LSD (23.5% vs. 13.5%, χ^2^_(1)_ = 9.80, *p* = 0.002).

**Table 4:** Reasons for not recommending specific psychedelics (*N* = 581)

Major themes	%
Characteristics of substance	70.4
Risks/safety concerns	30.1
Knowledge/Information is limited	21.3
Not therapeutic/No therapeutic indications/Lack of benefit*	2.8
Lack of Availability/Accessibility/Approval*	1.5
Depends on the person / Any can be OK in right circumstance*	1.5
Characteristics of substance	70.4
High potency/Too intense	52.4
Long duration	11.6
Synthetic/Not natural	6.8
Difficult to dose properly/precisely	6.8
Unpleasant/strange/weird/not representative of other psychedelics	6.8
Too unpredictable	4.6
Difficult to control purity/unreliable purity	3.3
Too rapid onset	2.3
Requires an experienced guide / facilitator	2.3
Long recovery time	1.5
Not psychedelic	1.0
Crash/empty feeling after use	1.0
Visually overwhelming	1.0
Difficult to prepare/administer	1.0
Lack of visuals	0.5
Too weak	0.3
Addictive	0.3
Creates vulnerability	0.3
Contraindications	
Mental health risks/safety concerns	19.2
Prone to adverse experiences (traumatizing, triggering, bad trips)	7.9
Anxiety/Fear	6.1
Exacerbation of existing mental health issues	1.5
Dissociative	1.3
Ego death/dissolution	1.3
Disorienting	1.0
Repressed memories may surface	1.0
Psychosis	0.5
Depression	
Physical health risks/safety concerns	14.9
GI upset	3.3
Purging	1.8
Toxic	1.3
Death/fatal overdose/risk of death from overdose	1.0
Heavy Body Load	0.8
Infertility	0.3
Knowledge/Information is limited	
No personal experience with substance	17.7
Unpleasant personal experience	1.0
Lack of research	0.5
No cultural traditions	0.5
Respect indigenous traditions by not stealing them	0.5

*No sub-themes

### Combinations recommended and not recommended for first-time psychedelic experiences

The most frequently recommended combinations included psilocybin and cannabis (12.6%), psilocybin and MDMA/MDA (9.1%), and LSD and MDMA/MDA (7.6%, Table 5). Less than a third of participants (29.3%) made any recommendation for combining psychedelics. Combinations recommended by fewer than 1% of participants (6 participants) are listed in Supplementary Table 1. Notably, 14.8% of participants recommended not mixing any psychedelics. When considering combining psychedelics with other drugs, the most frequent response was to not mix psychedelics with alcohol (20.8%) followed by not mixing psychedelics with stimulants (5.3%), antidepressants (4.0%), and narcotics/opiates (1.0%). Combinations not recommended by fewer than 1% of participants (6 participants) are listed in Supplementary Table 1.

**Table 5:** Most frequent recommendations for psychedelic combinations (*N* = 581)

Recommended combinations	%
Psilocybin and cannabis	12.6
Psilocybin and MDMA	9.1
LSD and MDMA	7.6
LSD and cannabis	5.2
Cannabis and other drugs	3.6
Psilocybin and LSD	2.9
Cannabis and MDMA	2.2
Ketamine and MDMA	1.9
Cannabis and Ketamine	1.4
LSD and Nitrous oxide	1.0
*No recommendation*	20.7
*Unsure, do not know*	10.2
Recommended to not combine	%
Do not mix any psychedelics	14.8
Psilocybin and LSD	1.5
Psilocybin and cannabis	1.0
*No recommendation*	14.3
*Unsure, do not know*	72
Do not mix with…	%
Alcohol	20.8
Stimulants	5.3
Antidepressants	4.0
Methamphetamine	1.5
Cocaine	1.4
Narcotics/opiates	1.0
Depends on the person	1.0

### Other recommendations

Most participants (83.5%) provided additional recommendations. The most common theme was mental preparation for the psychedelic experience (65.7%, Table 6), with frequent sub-themes including embracing the experience and whatever is encountered, learning about psychedelic experiences/substances being taken, and setting intentions for what to accomplish through the experience. About half of participants (50.9%) recommended preparing aspects of the environment for the psychedelic experience, including being with loved and trusted people, having a person who is experienced with psychedelics as a guide/sitter/facilitator, and being in a safe, familiar, and comfortable location. About a quarter of participants (24.2%) mentioned items to have on hand, such as music that will facilitate the trip, water (and hydrating often), and having a journal.

**Table 6:** Other recommendations (*N* = 581)

Theme	%
Mental preparation	65.7
Embrace the experience and whatever you encounter on your journey	30.3
Learn about psychedelic experiences/substance you are taking	13.9
Set intentions for what to accomplish through the experience	13.3
Clear time before and after for preparation and recovery	9.5
Prepare for anxiety/challenge, whatever you are feeling will go away	9.1
Meditation, breathwork, etc	7.2
Environment	50.9
Be with people you love/trust	23.8
Have a guide/sitter/facilitator	18.1
Be in a safe, familiar, and comfortable location	12.8
Be in nature	6.7
Have on hand	24.2
Music that will facilitate trip	9.5
Water/hydrate often	7.8
Journal	5.5
Comfortable clothing/blankets	3.2
Snacks (healthy, especially fresh fruit)	2.1
Eye masks	1.9
Trip killers	1.7
No phones	1.7
Prepare activities (drawing, creative activities)	1.7
Substance preparation	21.7
Start small (low doses)	15.2
Go slow (increase doses incrementally)	8.6
Know the source of your substance	3.2
Test your drugs	1.9
Diet preparation	7.6
Healthy food	1.9
Don’t drink alcohol before	1.9
Don’t eat too much before	1.0
Preparation for emergencies	5.3
Tell someone else your plans	2.7
Have an exit strategy	2.3
Ask for help when needed	0.4
Post-Experience Integration	4.4

One fifth (21.7%) of participants mentioned preparation related to the substance used, particularly starting with low doses and increasing doses incrementally. A few participants mentioned knowing the source of the substance (3.2%) and testing the substance (1.9%). A subset of participants (7.6%) advised dietary preparation, such as eating healthy food, not eating too much food, and not drinking alcohol before the experience. A few participants (5.3%) advised preparing for emergencies, such as telling plans to someone else, having an exit strategy, and asking for help when needed.

Overall, participants gave 14.2 unique pieces of advice on average across survey items (*SD* = 39.4, range: 0–392). Most participants (93.3%) gave at least one unique piece of advice; the median number was five. Participants who had taken a greater number of different psychedelics and participants who were younger in age provided more unique pieces of advice (Table 7). Once these variables were accounted for, there were no significant differences in the extent of advice by gender, ethnicity, education, income, frequency of psychedelic use in the past five years, or whether participants had experienced full doses of psychedelics or only microdoses.

**Table 7:** Results of linear regression predicting unique pieces of advice given (*N* = 581)

Coefficient	*B*	*SE*	*Beta*	*t*	*p*	B 95% CI LL	B 95% CI UL
(Constant)	24.94	5.96		4.18	< .001	13.23	36.65
Age in years	− 0.41	0.11	− .148	− 3.54	< .001	− 0.63	− 0.18
Total psychedelics taken	1.81	0.58	.130	3.12	.002	0.67	2.95

Education level in years, income in USD, White versus Non-White ethnicity, whether participants identified as a woman, whether participants identified as gender diverse (non-binary, transgender, etc.), frequency of psychedelic use in the past five years, and whether participants had experienced full doses of psychedelics or only microdoses did not contribute significant unique variance once these predictors were accounted for

## Discussion

To our knowledge, this is the first large-scale community-based study to compile recommendations for initial psychedelic experiences. Informed individuals are less likely to experience anxiety while using psychedelics [50]. Using the principles of community-based participatory research strengthened our initial survey design, ensuring relevance to individuals using psychedelics. This project recognizes the value of the collective knowledge within the psychedelic community and makes this knowledge accessible to the wider public. Participants who had experiences with specific substances were more likely to both recommend them (Psilocybin mushrooms or truffles, Cannabis, MDMA/MDA, LSD, and Ketamine) and NOT recommend them (Ayahuasca, DMT, 5-MeO-DMT, *Salvia divinorum* or salvinorin A, and LSD), indicating that these recommendations are in fact informed by lived experience. We recognize that the psychedelic community is not monolithic and that we recruited participants with a range of roles regarding psychedelics. All participants had personal experience with psychedelics and most reported professional roles (e.g., therapist, sitter, researcher, educator, activist), which may increase confidence in the resulting guidance. Those with professional roles may be providing advice based on content in the scientific literature, though the scarcity of peer-reviewed guidelines was the impetus for creating this project. Participants may also be sourcing advice from popular books and those from the underground press. Simultaneously, we note the need for continual education among those with psychedelic experience. The distribution of this work will aid in harm reduction and improve psychedelic experience outcomes due to the applicability of recommendations generated by the psychedelic community.

Individuals may find information about psychedelics on social media; however, social media platforms may censor this content [51] or eliminate fact checking, making the validity of content questionable. People who use psychedelics only somewhat trust information on psychedelics posted on social media, and consider articles published in peer-reviewed scientific journals as the most trusted source of information [52]. Internet websites are one of the most common sources of information on psychedelics [52], though they often contain harm reduction myths, such as advising people to take psychedelics alone, in a dark room or place, and drink orange juice to reduce the risk of adverse experiences [15].

### Convergence with current literature

The psychedelic harm reduction practices recommended by our survey participants converge with those given in qualitative studies and content in the broader scientific literature. The recommendation of psilocybin is consistent with the assessment of psilocybin by experts in substance use and abuse regarding its properties such as addiction potential, potential harm to individuals using, and potential harm to society [19, 20]. Extreme toxicity reports are more common with LSD or ayahuasca than psilocybin [53]. Those attending electronic music parties and festivals in Israel endorsed similar harm reduction practices, including researching about the substance before taking it, planning what substances to take, not mixing different substances (including alcohol), and being with trusted friends [34]. Psychedelic guides and facilitators often suggest specific medication regimens and, in some cases, diets to prevent serotonin syndrome [53]. Proper dosing is essential to avoid intense negative trips, which can lead to adverse mental health impacts such as hallucinogen-induced persistent psychosis [50]. Many sources recommended taking psychedelics with trusted individuals [15, 54].

In interviews, people using psychedelics in Norway reported rules they followed, including having a proper mindset and environmental setting, having knowledge of a substance and its effects, and developing a meditation practice to be able to observe and detach mindfully from thoughts [56]. These interviewees noted that their adverse experiences usually occurred when they disregarded these rules, and reported other recommendations such as talking to a loved one, sharing a plan, journaling, meditating, and being in a safe place with trusted people [56]. Another study reported that adverse outcomes were mostly due to overdosing, combining substances, lack of knowledge, police intervention, reckless attitudes, or substance misidentification [53]. A study examining mental health outcomes of naturalistic psychedelic use found that only 13% of participants reported negative mental health outcomes [5], which were associated with cannabis misuse and cigarette smoking [5].

### Set and setting

The primary focus of recommendations in the current literature is to have a conducive “set and setting.” "Set" refers to one's psychological state, including mood and intentions for using a psychedelic, as well as experiences and memories that are salient [55]. "Setting" refers to the environmental context in which the use occurs [55]. Participants in our survey noted that the outcomes of psychedelic experiences depended on set and setting, pieces of which aligned with the wide variety of advice across the literature on what constitutes a proper mindset. This includes specific advice such as limiting time on screens and social media, practicing meditation, having music playlists, and not having any prior commitments on the day of the trip [15]. Some sources advise to be in a good mood before tripping [15], whereas others argue that even negative experiences can be beneficial because they bring positive insights and help work through repressed traumas [56]. Some sources advise to set intentions or goals for the psychedelic experience, such as expanding thought, escaping routine, and seeking companionship and deeper human experiences [54]. Others argue that preconceived Western perspectives on psychedelics and society's obsession with personal goals makes it difficult for some individuals to detach fully and enjoy their experience [57]. Forstmann et al. [18] note that those who intended or expected to have a transcendent experience often reported one after psychedelic use, which they attributed to a self-fulfilling prophecy. Other research has found that the ability to surrender to the experience was the strongest predictor of having a mystical experience [57]. Our participants offered advice resembling the recommendations outlined above, suggesting that there are many considerations to keep in mind for first time psychedelic use.

In contrast to the increasingly standardized environments or “settings” in psychedelic clinical trials, there is no standard environment for naturalistic use, even for those with therapeutic intentions. These settings range from music festivals [27, 34] to natural environments providing solitude [51]. Our participants advised others to be in safe, familiar, and comfortable locations with trusted individuals, advice echoed in the existing literature [15, 34, 54, 56].

### The need for ongoing research and education

Few of our participants offered a comprehensive set of advice. This may result in part from the study methodology of an on-line survey with multiple components. More extensive advice might be obtained with other methods such as individual interviews. Still, few participants noted important issues such as serotonin syndrome even when asked specifically about harmful drug combinations. It is apparent that better education is necessary even for those with psychedelic.

experience. Even those with very extensive psychedelic experiences can have serious adverse events [58]. Many recommendations were also contradictory across participants (e.g., whether to combine substances or not), further research will be necessary to provide empirical support for many of these specific recommendations. We also acknowledge inconsistencies in opinions expressed in the peer-reviewed literature, as noted above.

## Limitations and recommendations for future research

Our study utilized a convenience sampling approach, which allowed us to include a large sample of participants. However, this sample may not be representative of the national or global population using psychedelics. Our sample was a conglomeration of participants with different roles regarding psychedelics, rather than a panel of experts in a single domain, such as substance abuse researchers [4, 19]. Although the most common roles participants cited regarding psychedelics were related to personal use (User, Personal Researcher, Psychonaut; See Table 1), most participants (54.7%) indicated having a role with expertise (Guide/Sitter, Therapist, Educator, Healthcare provider, Academic Researcher, Shamanic practitioner, Coach, Indigenous facilitator, Industry researcher, Trainer), with a third (34.4%) having two or more of these expert roles.

We did not ask participants to indicate the sources of their knowledge, e.g., personal experience or literature. We also conducted the survey in English. Recommendations may differ considerably in non-Western populations, especially in indigenous cultures with traditions of psychedelic practices. As the first study of its kind, we chose to use open-ended questions. We focused on recommendations for first experiences, and thus such advice may emphasize minimizing the risks of harm from psychedelic use. Maximizing benefits from psychedelic use would have a substantial relationship with minimizing risks but may also be a broader topic with substantial distinctions (e.g., continued use of psychedelics). The relationship between these aspects is an important question for future research which may also benefit from community-based approaches. The current project provides direction for more specific questions, e.g., choosing among response options. Future research would benefit from participant samples with greater national, racial/ethnic, and language diversity.

Further, because some participants had used psychedelics for many years, their views of what could lead to a positive first experience may be skewed by their current habits and use patterns, which may not translate to those using psychedelics for the first time. Recruitment from those participating in live and online psychedelic forums may bias the sample towards those who are more enthusiastic about psychedelics. However, the purpose of this project is to provide guidance for first-time psychedelic experiences. There is already an abundance of abstinence-based approaches recommending that people do NOT use psychedelics. It is also uncertain whether aspects of the harm reduction guidance offered apply more strongly to a therapeutic or recreational context, a topic that could be assessed in future research. As noted above, systematic empirical research regarding many of these topics is lacking. The guidance provided by our participants may be seen as a roadmap for future research programs. Randomized controlled trials examining the results of specific practices may be helpful but must be conducted carefully to avoid withholding essential safety information and harming participants.

## Conclusions

To our knowledge, this study represents the first large-scale community-based effort to compile recommendations for initial psychedelic experiences. Informed use and harm reduction practices can significantly enhance the safety and positive outcomes of psychedelic experiences. The convergence of our participants' recommendations with existing literature highlights the value of community knowledge in guiding safe and beneficial psychedelic use. However, the need for ongoing education and more comprehensive advice remains evident. Future research should aim to include more diverse participant samples and explore additional methods to gather detailed recommendations. By continuing to integrate community insights with scientific research, we can better support individuals in their psychedelic journeys and contribute to the broader understanding of these substances.

## Supplementary Information

Additional file.

## Data Availability

Materials will be available upon reasonable request.

## Abbreviations

5-MeO-DMT: 5-Methoxy-*N*,*N*-dimethyltryptamine
AIDS: Acquired immunodeficiency syndrome
DMT: *N*,*N*-dimethyltryptamine
FDA: Food and Drug Administration
HIV: Human immunodeficiency virus
LSD: Lysergic acid diethylamide
MDMA: 3,4-Methylenedioxymethamphetamine
MDA: 3,4-Methylenedioxyamphetamine
