# Supplementary material for: Best practices for first psychedelic experiences: harm reduction advice from the psychedelic community
Source: Harm Reduct J. 2025 Nov 25;22:191. doi: 10.1186/s12954-025-01337-2 (PMC12648905; doi:10.1186/s12954-025-01337-2)
Supplement: Supplementary file 1 — Additional file. [file 12954_2025_1337_MOESM1_ESM.docx]

**Table S1** Infrequent recommendations for psychedelic combinations (*N* = 581)

| **Recommended combinations** | ***n*** |
| --- | --- |
| Ketamine and LSD | 5 |
| Ketamine and Psilocybin | 5 |
| DMT and LSD | 4 |
| DMT and Psilocybin | 4 |
| Mescaline and Psilocybin | 4 |
| Nitrous oxide and Psilocybin | 4 |
| Cannabis and DMT | 3 |
| Cannabis and Mescaline | 3 |
| Dextromethorphan and LSD | 3 |
| LSD and Mescaline | 3 |
| LSD and Synthetic phenethylamines | 3 |
| MDMA/MDA and Synthetic phenethylamines | 3 |
| 5-MeO-DMT and MDMA | 2 |
| *Amanita muscaria* and Cannabis | 2 |
| Cannabis and Nitrous oxide | 2 |
| 5-MeO-DMT and Iboga/Ibogaine | 1 |
| 5-MeO-DMT and *Salvia divinorum* | 1 |
| *Amanita muscaria* and LSA | 1 |
| *Amanita muscaria* and LSD | 1 |
| *Amanita muscaria* and MDMA/MDA | 1 |
| *Amanita muscaria* and Mescaline | 1 |
| *Amanita muscaria* and Psilocybin | 1 |
| Ayahuasca and Psilocybin | 1 |
| Cannabis and Dextromethorphan | 1 |
| Dextromethorphan and Mescaline | 1 |
| Dextromethorphan and Psilocybin | 1 |
| DMT and Ketamine | 1 |
| DMT and Mescaline | 1 |
| DMT and *Salvia divinorum* | 1 |
| Ketamine and Mescaline | 1 |
| Ketamine and Nitrous oxide | 1 |
| Ketamine and Synthetic phenethylamines | 1 |
| LSA and MDMA/MDA | 1 |
| LSA and Mescaline | 1 |
| MDMA/MDA and Mescaline | 1 |
| MDMA/MDA and Nitrous oxide | 1 |
| Mescaline and Nitrous oxide | 1 |
| Psilocybin and *Salvia divinorum* | 1 |
|  |  |
| **Recommended to not combine** | ***n*** |
| MDMA/MDA and Psilocybin | 4 |
| Ayahuasca and Cannabis | 2 |
| Cannabis and Ketamine | 2 |
| DMT and LSD | 2 |
| Ketamine and LSD | 2 |
| LSD and MDMA/MDA | 2 |
| 5-MeO-DMT and Psilocybin | 1 |
| *Amanita muscaria* and Dextromethorphan | 1 |
| Ayahuasca and 5-MeO-DMT | 1 |
| Ayahuasca and MDMA/MDA | 1 |
| Cannabis and DMT | 1 |
| Dextromethorphan and MDMA/MDA | 1 |
| Dextromethorphan and Synthetic phenethylamines | 1 |
| DMT and MDMA/MDA | 1 |
| DMT and *Salvia divinorum* | 1 |
| Iboga/Ibogaine and Ketamine | 1 |
| Iboga/Ibogaine and MDMA/MDA | 1 |
| Ketamine and MDMA/MDA | 1 |
| LSD and *Salvia divinorum* | 1 |
| **Do not mix with…** | ***n*** |
| Sedatives/Tranquilizers | 5 |
| Antipsychotics | 3 |
